# Multiferroic Properties of Co, Ru, and La Ion Doped KBiFe_2_O_5_

**DOI:** 10.3390/ma17010001

**Published:** 2023-12-19

**Authors:** Angel Apostolov, Iliana Apostolova, Julia Wesselinowa

**Affiliations:** 1 Department of Physics, Faculty of Hydrotechnics, University of Architecture, Civil Engineering and Geodesy, Hristo Smirnenski Blvd. 1, 1046 Sofia, Bulgaria; angelapos@abv.bg; 2 Faculty of Forest Industry, University of Forestry, Kl. Ohridsky Blvd. 10, 1756 Sofia, Bulgaria; inaapos@abv.bg; 3 Faculty of Physics, Sofia University “St. Kliment Ohridski”, J. Bouchier Blvd. 5, 1164 Sofia, Bulgaria

**Keywords:** ion doped KBiFe_2_O_5_, multiferroic properties, microscopic model, Green’s function theory

## Abstract

The magnetic, electric, dielectric, and optical (band gap) properties of ion doped multiferroic KBiFe${}_{2}$O${}_{5}$ (KBFO) have been systematically investigated utilizing a microscopic model and the Green’s function theory. Doping with Co at the Fe site and Ru at the Bi site induces changes in magnetization, coercive field, and band gap energy. Specifically, an increase in magnetization is observed, while the coercive field and band gap energy decrease. This behavior is attributed to the distinct ionic radii of the doped and host ions, leading to alterations in the exchange interaction constants. The temperature dependence of the polarization *P* reveals a distinctive kink at the Neel temperature $T_{N}$, which shifts to higher temperatures with an increase in the applied magnetic field *h*. Furthermore, doping with Ru and La leads to an increase in polarization. The temperature dependence of the dielectric constant exhibits two peaks at the Neel temperature $T_{N}$ and the Curie temperature $T_{C}$. Notably, these peaks diminish with increasing frequency. Additionally, the dielectric constant demonstrates a decrease with the rise in the applied magnetic field *h*. This study sheds light on the intricate interplay between ion doping, structural modifications, and multifunctional properties in KBFO, offering valuable insights into the underlying mechanisms governing its behavior across various physical domains.

## 1. Introduction

Several multiferroic materials, such as KBiFe${}_{2}$O${}_{5}$ (KBFO), are currently under intense scrutiny for their potential applications in solar cells. This interest stems from their unique combination of ferroelectricity and magnetism, as well as a notably low band gap. The multiferroicity of KBFO was first reported by Zhang et al. [1,2]. KBFO belongs to the brownmillerite class of crystal structure at room temperature with orthorhombic space group $P2_{1}cn$. KBFO exhibits both ferroelectric and magnetic transitions at temperatures ∼780 K and ∼550 K, respectively. It qualifies as a room-temperature multiferroic compound, possessing a narrow band gap of 1.6 eV [1,3]. The suitability of brownmillerites for photovoltaic and photocatalytic applications is attributed to their low band gap. The magnetic properties of KBFO arise from the presence of Fe atoms, as K and Bi are nonmagnetic. The observed weak ferromagnetism is a consequence of the high spin states of Fe${}^{3+}$ in the FeO${}_{4}$ tetrahedron, resulting from G-type canted antiferromagnetic ordering of Fe ions. Ferroelectricity in KBFO is attributed to displacements of K${}^{+}$ and Bi${}^{3+}$ ions. Magnetoelectric, magnetodielectric, and optical properties of bulk KBFO [4,5,6,7,8] and KBFO thin films showing well-saturated polarization hysteresis loops [9,10,11] have been considered in the last few years. The band gap value of KBFO thin films with orthorhombic structure is found to be 1.67 eV [10], i.e., it is larger than in bulk KBFO and is appropriate for visible light absorption. The magnetic properties of nano-sized KBFO were studied theoretically using Monte Carlo simulations by Housni et al. [12]. These studies have outlined different exchange coupling interaction constants between the Fe atoms, considering the angles between Fe1-O-Fe2. The existence of oxygen atoms induces a double exchange interaction, contributing to the overall magnetic behavior of the material.

The doping effects with different ions (La, Y, Co, Ho, Ru, and Al) on structural, magnetic, electrical, and optical properties of KBFO have also been investigated [13,14,15,16,17,18]. The doping ions can change the properties of the compound. By doping with Co or Al (at the Fe site) and with La, Y or Ru (at the Bi site), the magnetization increases (attributed to the modification of spin canting), whereas the band gap decreases [13,15,16,18]. The decrease in band gap can be due to the tilting of the Fe-O tetrahedral structure of Co-doped KBFO. An increase in magnetization, band gap energy, and photocatalytic efficiency was observed for Y ion-doped KBFO by Rai et al. [14]. Chandrakanta et al. [15] have contributed to the understanding of the dielectric properties of Co-doped KBFO, demonstrating an enhancement in these properties. The intricate interplay between the dopant ions and the various properties of KBFO highlights the versatility of doping as a strategy to tailor the material’s characteristics for specific applications. In comparison to KBFO, the frequency-dependent dielectric constant and dielectric loss of Co-doped KBFO exhibit a decrease at room temperature. This reduction can be attributed to the reduction in oxygen migration and alterations in response to vibrational modes within the sample. The introduction of Ru as a dopant not only increases the magnetization and the dielectric properties, but also leads to a reduction in the band gaps [16]. Ru is anticipated to minimize leakage current, thereby significantly transforming the structural and magnetoelectric properties of KBFO. This transformation facilitates interaction between 4d (Ru) and 3d (Fe) electrons, potentially causing substantial changes in the electronic and magnetic ground states. Additionally, Ru doping in various ferroelectric systems has been shown to elevate polarization, magnetization, and dielectric properties while concurrently reducing the optical band gaps. La doping proves to enhance the magnetic properties of the compound, with maximum magnetization experiencing an increase upon La doping [13]. However, a noteworthy reduction in coercivity is observed in La doped samples. Furthermore, La doping results in a narrowing of the optical band gap. The ferroelectric properties in composite KBFO-polymer thin films are also enhanced by the incorporation of La, evidenced by a reduction in leakage current and well-saturated polarization hysteresis loops [19]. This suggests that doping, particularly with elements like Ru and La, serves as a versatile approach to tailor the properties of KBFO, making it adaptable for various applications.

Raman peaks of Co-doped KBFO shifted by 3 cm${}^{-1}$ to lower wave numbers compared to those of pure KBFO [20]. This alteration in Raman active modes is attributed to changes in the bond angles and bond lengths within the Fe-O tetrahedral structure. The modification is a response to the presence of oxygen deficiency induced by Co doping.

It is noteworthy to mention that, as of now, a theoretical explanation for the multiferroism observed in both pure and ion doped KBFO has not been established. That is why we intend to employ a microscopic model and Green’s function theory in order to study the magnetic, electric, dielectric, and optical properties of both pure and ion-doped KBFO. In order to account for the influence of ion doping on the magnetic and ferroelectric properties, we modified the Heisenberg model and transverse Ising model (introduced by de Gennes [21]), respectively. For the magnetoelectric coupling, we assumed a biquadratic one. The subsequent results will be compared with existing experimental data, aiming to provide further understanding of the complex interplay between structural modifications and multifunctional properties of KBFO.

## 2. The Model and the Green’s Functions

KBFO has a structure derived from the double perovskites (A’A”B${}_{2}$O${}_{6}$), but with oxygen deficiency. It is classified as having a brownmillerite structure with a new arrangement between Fe and O (tetrahedral instead of octahedral). KBFO is usually orthorhombic at room temperature and pressure; however, it transforms into a monoclinic structure at high temperatures and pressures. The monoclinic structure has more interesting multiferroic and electronic properties for photovoltaic applications. For the Pc/n monoclinic structure, the lattice parameters are *a* = 7.87637 $\dot{A}$, *b* = 5.97527 $\dot{A}$, and *c* = 5.77440 $\dot{A}$. For the P${}_{21}$cn orthorhombic structure, the lattice parameters are *a* = 7.98415 $\dot{A}$, *b* = 11.81928 $\dot{A}$, and *c* = 5.73934 $\dot{A}$. KBFO shows both ferroelectric transition and magnetic transition above room temperature at ∼780 K and at ∼560 K, respectively. So, it can be considered as a room temperature multiferroic compound.

The multiferroic properties of KBFO can be expressed by using the following Hamiltonian:

(1) $$H=H_{m}+H_{e}+H_{me}.$$

The magnetic properties of the ion doped KBFO are described by a modified Heisenberg model:

(2) $$\begin{array}{ccc} H_{m} & = & -\sum_{i,j}\left(1-x\right)J_{ij}^{Fe-Fe}S_{i}^{Fe}\cdot S_{j}^{Fe}-\sum_{i,j}x\left(x^{\prime }\right)J_{ij}^{Fe-DI}S_{i}^{Fe}\cdot S_{j}^{DI}\\ & - & \sum_{i}D_{i}{\left(S_{i}^{zFe}\right)}^{2}-g\mu_{B}h\cdot \sum_{i}S_{i}^{Fe}, \end{array}$$

where $S_{i}^{Fe,DI}$ is a Heisenberg spin operator of the Fe ion or doping ion (DI) at the site *i*. $J_{ij}^{Fe-Fe}$ (taking into account the nearest (nn) and next-nearest (nnn) neighbours) and $J_{ij}^{Fe-DI}$ are the exchange interaction constants between the Fe-Fe and Fe-DI ions, respectively, *x* and $x^{\prime }$ are the concentrations of the substituted ions at the Fe or Bi site, respectively, $D_{i}$ is the single-ion anisotropy constant, and *h* is an external magnetic field.

The ferroelectric properties of KBFO caused by displacements of the K${}^{+}$ and Bi${}^{3+}$ ions can be described by the Ising model in a transverse field:

(3) $$H_{e}=-\Omega \sum_{i}B_{i}^{x}-\frac{1}{2}\sum_{ij}\left(1-x^{\prime }\right)J_{ij}^{\prime }B_{i}^{z}B_{j}^{z},$$

where $B_{i}^{x}$, $B_{i}^{z}$ are pseudo-spin operators, $J_{ij}^{\prime }$ is the nearest-neighbor exchange pseudo-spin interaction, Ω is the tunneling frequency, and $x^{\prime }$ denotes the doping concentration by substitution at the Bi site.

The magnetoelectric term that couples the two subsystems could be given by:

(4) $$H_{me}=-g\sum_{ijkl}B_{i}^{z}B_{j}^{z}S_{k}\cdot S_{l}.$$

We assume a quadratic magnetoelectric coupling *g* in KBFO because of the big difference between the ferroelectric Curie temperature $T_{C}$ and the magnetic phase transition temperature $T_{N}$, $T_{C}>>T_{N}$. Moreover, the biquadratic nature of the magnetoelectric coupling is also confirmed using the Landau free theory [15].

The spontaneous magnetization $M=\langle S^{z}\rangle$ is calculated as

(5) $$M=\langle S^{z}\rangle =\frac{1}{N}\sum_{ij}\left[\left(S+0.5\right)\coth\left[\left(S+0.5\right)\beta E_{mij}\right)]-0.5\coth\left(0.5\beta E_{mij}\right)\right],$$

where *S* is the spin value, $\beta =1/k_{B}T$. $E_{mij}$ are the spin excitations calculated from the spin Green’s function $G_{ij}=\ll S_{i}^{+};S_{j}^{-}\gg$:

(6) $$E_{mij}=\frac{\langle \left[\left[S_{i}^{+},H\right],S_{j}^{-}\right]\rangle }{\langle \left[S_{i}^{+},S_{j}^{-}\right]\rangle }.$$

The spontaneous polarization *P* is calculated from the poles of the Green’s function:

(7) $${\tilde{G}}_{ij}=\ll B_{i}^{+};B_{j}^{-}\gg$$

as

(8) $$P=\frac{1}{2N}\sum_{i}\tanh\frac{E_{fi}}{2k_{B}T}.$$

$E_{fi}$ is the pseudo-spin wave energy.

$H_{el}$ is the Hamiltonian of the conduction band electrons:

(9) $$H_{el}=\sum_{ij\sigma }t_{ij}c_{i\sigma }^{+}c_{j\sigma }.$$

$t_{ij}$ is the hopping integral, $c_{i\sigma }^{+}$ and $c_{i\sigma }$ are Fermi-creation and -annihilation operators.

The s-d coupling term $H_{m-el}$ reads

(10) $$H_{m-el}=\sum_{i}I_{i}S_{i}s_{i}.$$

Here *I* is the s-d interaction constant and $s_{i}$ are the spin operators of the conduction electrons.

The difference between the valence and conduction bands determines the band gap energy $E_{g}=\omega^{+}\left(k=0\right)-\omega^{-}\left(k=k_{\sigma }\right)$. The electronic energies

(11) $$\omega_{ij\sigma }^{\pm }=\epsilon_{ij\sigma }-\frac{\sigma }{2}I\langle S^{z}\rangle$$

are observed from the Green’s functions $g_{ij\sigma }=\ll c_{i\sigma };c_{j\sigma }^{+}\gg$, σ=±1. $\epsilon_{ij\sigma }$ is the conduction band energy in the paramagnetic state, *I*—the s-d interaction constant, and $\langle S^{z}\rangle$—the magnetization.

The dielectric function ϵ is obtained from the following equation [22]:

(12) $$\left({\left(\Lambda /\left(\epsilon \left(E\right)-1\right)\right)}_{\alpha \beta }+\Lambda \frac{k_{\alpha }k_{\beta }}{k^{2}}\right){\tilde{G}}^{\beta \gamma }\left(E\right)=\delta_{\alpha \gamma };\;\;\Lambda =4\pi Z^{2}/v.$$

*Z* is the electron charge and *v*—the volume. In order to obtain the dielectric function ϵ we have calculated the longitudinal Green’s function ${\tilde{G}}^{zz}\left(E\right)=\langle \langle B_{i}^{z};B_{j}^{z}\rangle \rangle$.

In summary, we have shown how we can observe the macroscopic quantities (magnetization, polarization, dielectric constant, band gap—Equations (5), (8), (11) and (12)) from the corresponding Green’s functions (see also Equation (6)). In order to observe these quantities, we need Hamiltonians, which are defined for the magnetic and ferroelectric subsystems (taking into account different interactions), and the coupling term between them (see Equations (1)–(3)). So, using these theoretical approaches, we can obtain macroscopic quantities which are explained on a microscopic level.

## 3. Numerical Results and Discussion

The new multiferroic compound KBFO has a brownmillerite structure type (A${}_{2}$B${}_{2}$O${}_{5}$) with a tetrahedral instead of octahedral arrangement between Fe and O [1,2]. FeO${}_{6}$ octahedra are absent compared with the normal brownmillerites. KBFO contains only tetrahedral Fe${}^{3+}$ in a [Fe${}_{2}$O${}_{3}$] block.

The following model parameters are used for the numerical calculations of KBFO: $J_{nn}^{Fe-Fe}$ = 112 K, $J_{nnn}^{Fe-Fe}$ = −52.5 K, $D^{Fe}$ = −20 K, $J^{\prime }$ = 715 K, Ω = 2 K, *I* = 30 K, *t* = 11 K, *g* = 50 K, S(Fe${}^{3+}$) = 5/2, and *S*(pseudo-spin) = 1/2.

We will give a short description of how we observe the model parameters. By using the values of the experimentally known Neel temperature $T_{N}$ and asymptotic Curie–Weiss temperature $\Theta_{W}$, the nearest neighbors $z_{1}$ and the next nearest neighbors $z_{2}$ the exchange interactions between the Fe ions are estimated from the following molecular field relations:

(13) $$\begin{array}{ccc} \Theta_{W} & = & 2S\left(S+1\right)\left(z_{1}J^{nn}+z_{2}J^{nnn}\right)/\left(3k_{B}\right), \end{array}$$

(14) $$\begin{array}{ccc} T_{N} & = & 2S\left(S+1\right)\left(-z_{1}J^{nn}+z_{2}J^{nnn}\right)/\left(3k_{B}\right). \end{array}$$

The value for the exchange interaction constant $J^{\prime }$ is estimated from the expression in mean-field theory $J^{\prime }=3k_{B}T_{C}/\left(zS\left(S+1\right)\right)$, where *z* is the number of nearest neighbors, *S*—the spin value, $T_{C}$—the Curie temperature, and $k_{B}$—the Boltzmann constant. The tunneling frequency Ω is observed from the expression for the pseudo-spin wave energy by very high temperatures: $E_{fi}=2\Omega$.

### 3.1. Temperature Dependence of the Magnetization for Pure KBFO

**Figure 1:**
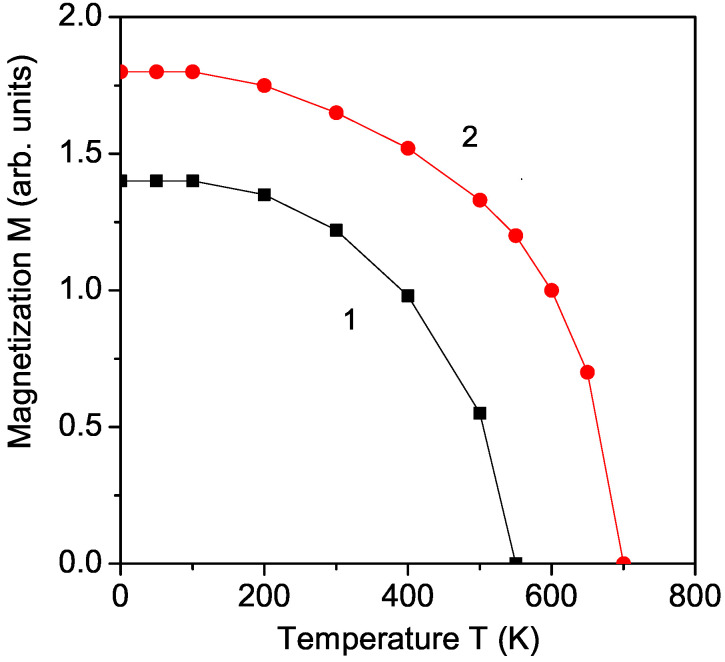
(Color online) Temperature dependence of the magnetization *M* for (1) pure KBFO and (2) Co doped KBFO (*x* = 0.05, $J_{d}=1.2J$).

At first, the temperature dependence of the spontaneous magnetization *M* for pure KBFO is calculated. The result is presented in Figure 1, curve 1. *M* is reduced with a rising temperature and vanishes at the magnetic phase transition temperature $T_{N}$∼550 K. By doping KBFO with Co ions at the Fe site, there appears a compressive strain, due to the different ionic radii between the host Fe${}^{3+}$ ion (*r* = 0.64 $\dot{A}$) and the doping Co${}^{3+}$ ion (*r* = 0.6 $\dot{A}$) [20]. The Co doping changes the interatomic distances of the Fe-O, Fe-Bi and Bi-O bonds, which are shortened. The exchange interaction constant $J(r_{i}-r_{j})$ is inversely proportional to the distance between the spins, i.e., to the lattice parameters. Chadrakanta et al. [20] reported a decrease in the lattice parameters by increasing the Co doping concentration. This means that the exchange interaction constants at the doping states, denoted as $J_{d}$, are larger in comparison to the undoped ones, denoted as *J*, $J_{d}>J$. This leads to an increase in the magnetization *M* in Co-doped KBFO (see Figure 1, curve 2), in coincidence with the experimental data of Chandrakanta et al. [15]. It can be seen that the Neel temperature $T_{N}$ also increases when increasing the concentration of the dopant Co. Let us emphasize that $T_{N}$ can reach a certain *x* value and Curie temperature $T_{C}$ and can exceed it. A similar enhanced magnetization *M* is observed by Sahoo et al. [18] in Al-doped KBFO, where again a compressive strain appears and could be explained by our model and method.

### 3.2. Ru Ion Doping Effects on Magnetization and Coercive Field in KBFO

**Figure 2:**
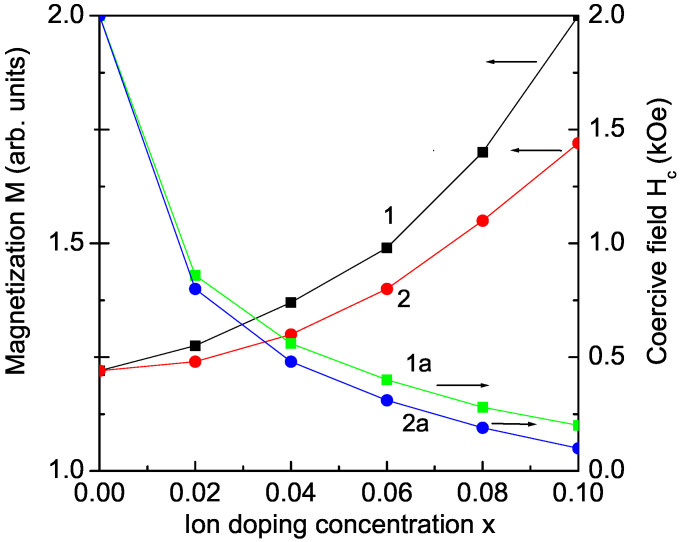
(Color online) Dependence of the magnetization *M* (1, 2) and coercive field $H_{c}$ (1a, 2a) on the doping concentration for Ru ($J_{d}^{\prime }=1.2J^{\prime }$) and La ($J_{d}^{\prime }=1.1J^{\prime }$) doping, respectively, *T* = 300 K.

Ion doping at the Bi site can also lead to increased spontaneous magnetization *M*. We consider the case of Ru ion doping. The interaction between 4d (Ru) and 3d (Fe) electrons changes the electronic and magnetic properties. The lattice parameters reported by Bitra et al. [16] decrease with increasing Ru ion substitution due to the difference between the Bi${}^{3+}$ (*r* = 1.17 $\dot{A}$) and Ru${}^{3+}$ (*r* = 0.82 $\dot{A}$) ionic radii, where the radius of the doping Ru ion is smaller than that of the host Bi ion. A compressive strain appears, i.e., $J_{d}>J$ and $J_{d}^{\prime }>J^{\prime }$, which enhances the spontaneous magnetization *M*. This is presented in Figure 2, curve 1. It must be mentioned that the coercive field $H_{c}$ is reduced when increasing Ru doping ions, which can be seen in Figure 2, curve 1a. A similar behavior of *M* and $H_{c}$ is reported also in La ion-doped KBFO by Rai et al. [13], which is obtained with our model and method too (see Figure 2, curves 2, 2a). The La${}^{3+}$ ions lead to a modification of the spin canting and an increase in the spontaneous magnetization. The reported increase in *M* in Ho-doped KBFO could also be explained within our model [23].

### 3.3. Ru and La Ion Doping Effects on the Polarization in KBFO

**Figure 3:**
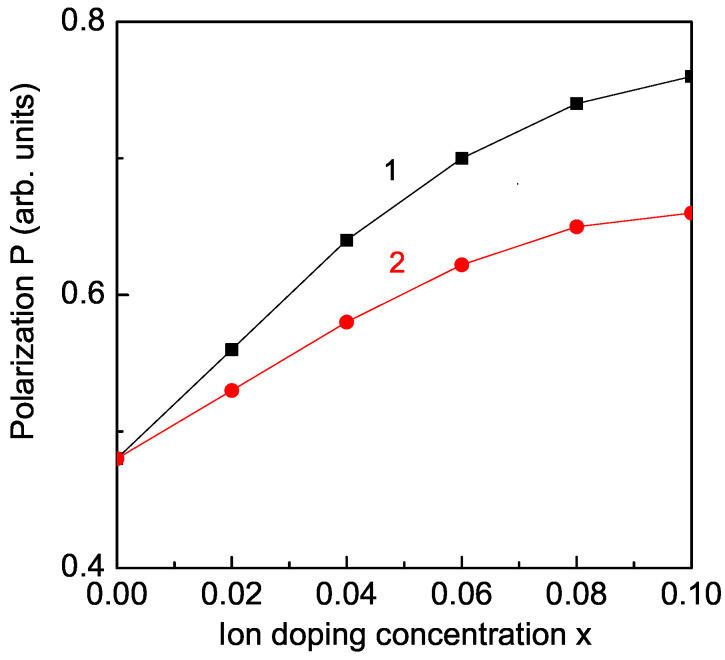
(Color online) Concentration dependence of the polarization *P* for *T* = 300 K and different doping ions: (1) Ru ($J_{d}^{\prime }=1.2J^{\prime }$), (2) La ($J_{d}^{\prime }=1.1J^{\prime }$).

The spontaneous polarization *P* is studied from Equation (7) in Ru doped KFBO, which is enhanced in comparison with the undoped KFBO compound (see Figure 3, curve 1). This is in coincidence with the experimental results of Bitra et al. [16]. La ion doping also enhances the polarization *P*, as can be seen in Figure 3, curve 2. This is obtained in La-doped KBFO by Rai et al. [13].

### 3.4. Temperature Dependence of the Polarization for Pure KBFO

**Figure 4:**
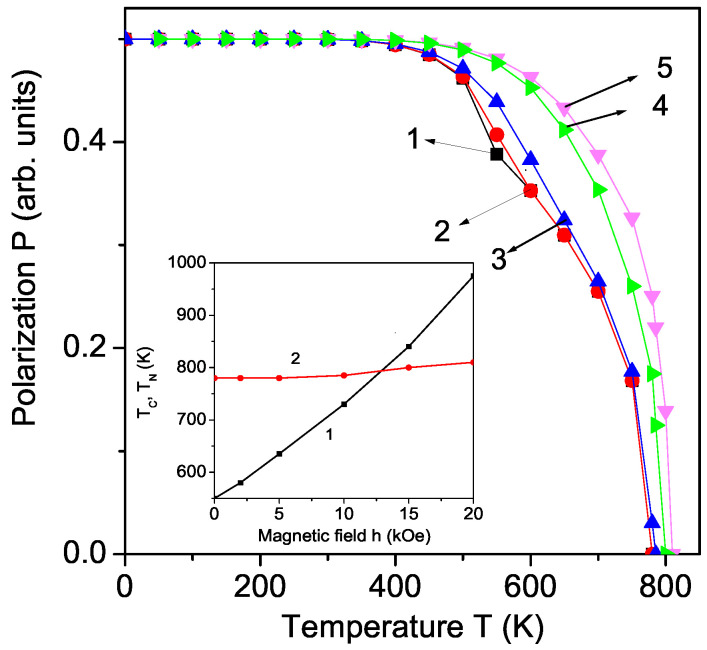
(Color online) Temperature dependence of the polarization *P* of pure KBFO for different magnetic field *h* values: 0 (1), 2 (2), 10 (3), 15 (4), 20 (5) kOe. Inset: Magnetic field dependence of the Neel $T_{N}$ (1) and Curie $T_{C}$ (2) temperature for pure KBFO.

The temperature dependence of the polarization *P* for pure KBFO is also calculated (see Figure 4, curve 1). *P* decreases with raising temperature and goes to zero at the Curie temperature $T_{C}$ = 780 K. From the figure, it can be seen that there is a kink in the curve at the magnetic phase transition temperature $T_{N}$ = 550 K. This confirms the multiferroic behavior of the compound.

### 3.5. Magnetic Field Dependence of the Polarization and the Neel Temperature for Pure KBFO

Furthermore, the multiferroic order demonstrates a magnetic field control of the electric polarization [24,25,26]. Applying an external magnetic field *h* the kink in P(T) is smaller and shifted to higher *T* values (see Figure 4, curves 2 and 3) because the Neel temperature $T_{N}$ increases (see inset in Figure 4). For *h* = 2, 5, 10, 15, 20 kOe we observe a Neel temperature $T_{N}$∼ 580, 635, 730, 840, 975 K, respectively. It can be seen that for large *h* values (for example > 15–20 kOe) $T_{N}$ is larger than $T_{C}$ and the kink in P(T) vanishes. Applying a magnetic field, the multiferroic phase is wider. For *h* = 10, 15 and 20 kOe, $T_{C}$ is 785, 800 and 810 K, i.e., it is also increased (see inset in Figure 4).

### 3.6. Temperature and Frequency Dependence of the Dielectric Constant for Pure KBFO

**Figure 5:**
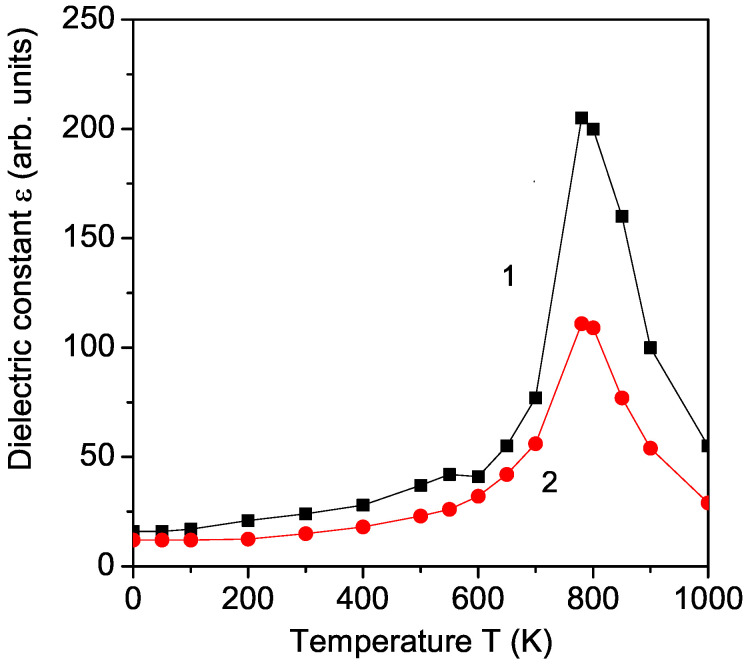
(Color online) Temperature dependence of the dielectric constant ϵ of pure KBFO for different frequencies *f*: 5 (1); 10 (2) kHz.

As a next, from Equation (12) we have calculated the real part of the dielectric constant ϵ in pure KBFO for different temperatures and frequencies. The temperature dependence of ϵ is presented in Figure 5, curve 1. There is a small anomaly around the Neel temperature $T_{N}$ ∼ 550 K and a large peak at the Curie temperature $T_{C}$∼ 780 K. These two peaks supported the multiferroic behavior of KBFO. Let us emphasize that Vavilapalli et al. [7] observed the large peak at $T_{C}$ in the dielectric constant ϵ of KBFO, but not the anomaly at the Neel temperature $T_{N}$. Chandra et al. [23] reported that the study of the temperature dependence of the dielectric constant of Ho-doped KBFO shows two transitions, at $T_{N}$ and $T_{C}$. The temperature dependence of ϵ of Al doped KBFO was investigated for *T* = 300–773 K by Sahoo et al. [18] which exhibits a small anomaly at the Neel temperature $T_{N}$∼550 K.

The frequency dependence of the dielectric constant ϵ is also considered (see Figure 5, curve 2). At lower frequencies, ϵ shows higher values, which is due to the contribution of space charge, dipolar, and electronic polarizations [2]. With increasing frequency, the dielectric constant ϵ is reduced due to the decrease in the space charge effect. The small anomaly at the Neel temperature $T_{N}$ disappears, and the peak at the Curie temperature $T_{C}$ decreases and is wider. For large frequency values, the peak at $T_{C}$ disappears, too.

### 3.7. Magneto-Dielectric Effect in Pure KBFO

**Figure 6:**
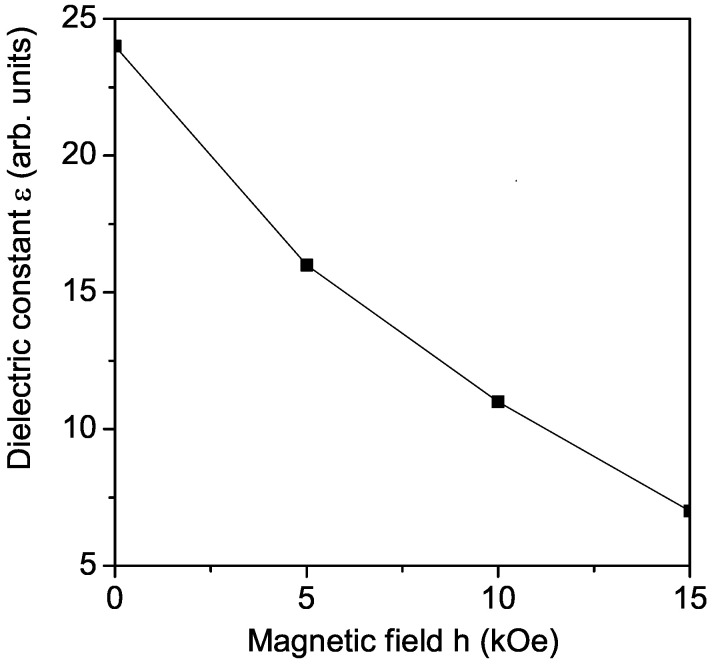
Magnetic field dependence of the dielectric constant ϵ of pure KBFO for *T* = 300 K, *f* = 5 kHz.

In order to consider the magneto-dielectric effect, the dielectric constant ϵ for pure KBFO in dependence of the external magnetic field *h* is calculated. The result is presented in Figure 6. The dielectric constant ϵ decreases with increasing *h*. The magneto-dielectric coupling MD(%)=(ϵ(h)−ϵ(0))/ϵ(0) is negative, where ϵ(h) and ϵ(0) are the dielectric constant with and without magnetic field, respectively. This is in agreement with the experimental data of Khan et al. [27] and Chandra et al. [15,17].

### 3.8. Ru and Co Ion Dependence of the Band Gap Energy for KBFO

**Figure 7:**
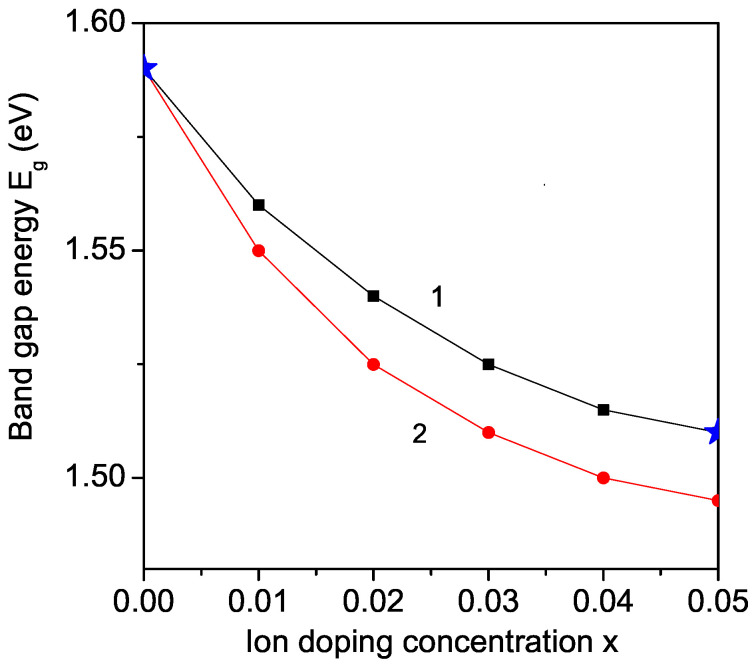
(Color online) Concentration dependence of the band gap energy $E_{g}$ of KBFO for *T* = 300 K and different doping ions: Co (1); Ru (2). The blue stars are the experimental values from Ref. [20].

Finally, from Equation (11) the band gap energy $E_{g}$ is studied as a function of the ion doping. Let us emphasize that values around 1.6 eV for orthorhombic structures (which are here considered) [1,9] and around 1.7 eV for monoclinic structures [2,13] are reported. The low band gap behavior in KBFO is caused by the FeO${}_{4}$ tetrahedron structure with small crystal field splitting energy [13]. The results for Co and Ru ion doped KBFO are shown in Figure 7. It can be seen that the band gap energy $E_{g}$ decreases with increasing ion dopant, in agreement with the experimental data [13,16,20]. This decrease in $E_{g}$ can be attributed to the tilting of the Fe-O tetrahedral structure of ion-doped KBFO. Moreover, by the ion doping with Co or Ru the magnetization *M* increases, and from Equation (11), it can be seen that this leads to reduced $E_{g}$ values. In principle, the optical band gap $E_{g}$ increases or decreases according to the density of localized states, low or high, respectively. The higher the localized state, the lower the optical band gap $E_{g}$ or vice versa, the lower the localized state, the higher the band gap. Thus, Co substitution at the Fe site of KBFO leads to the enhancement of its optical properties with the reduction in its band gap energy. Moreover, by Co doping there is a good quantitative agreement with the experimental data of Chandrakanta et al. [20], which is evidence that the proposed model and the used method of the Green’s functions are appropriate. A decrease in the band gap energy $E_{g}$ is reported also by doping with other ions, for example Al [18], and La [13]. It must be mentioned that Rai et al. [14] reported an increase in magnetization *M* and band gap energy $E_{g}$ for Y ion doped KBFO. We would observe an increase in the magnetization *M* because the ionic radius of Y${}^{3+}$ (*r* = 1.04 $\dot{A}$) is smaller than that for Bi${}^{3+}$ (*r* = 1.17 $\dot{A}$), i.e., we have again a compressive strain. But contrary to the result of Rai et al. [14], we would obtain a decrease in the band gap energy $E_{g}$ by increasing the Y dopant.

It could be mentioned, as it was shown previously through the connection between the lattice parameters, the lattice structure and the magnetization with the band gap energy *E_g_*, that an applied magnetic field *h* could also affect *E_g_*. Increasing the magnetic field *h* would lead to decreasing of the band gap energy *E_g_* in pure KBFO. Through Co or Ru ion doping, the lattice parameters decrease and *E_g_* decreases, additively. Unfortunately, there are no experimental studies for *E_g_*(*h*) in KBFO.

Let us emphasize that by replacing the Fe ions with ions that have larger ionic radii, we would observe a tensile strain, the exchange interaction constants would decrease and the magnetization *M* would be reduced, whereas the band gap energy *E_g_* would be enhanced.

## 4. Conclusions

Utilizing a microscopic model and Green’s function theory, we have investigated the magnetic, electric, dielectric, and optical (band gap) properties of ion-doped KBFO. KBFO stands out as an intriguing multiferroic compound, characterized by the coexistence of ferroelectric and ferromagnetic phases and by a narrow band gap. The ion doping leads to the appearance of different strains; to a change in the lattice parameters, which, due to the difference between the ionic radius of the doping and host ion, leads to changes in the exchange interaction parameters; and of the observed macroscopic properties—to increase or decrease in dependence on the different strain. In Co- and Ru-doped KBFO, we observed an increase in magnetization accompanied by a decrease in both coercive field and band gap energy. This phenomenon is attributed to the differing ionic radii of the doped and host ions, which leads to different exchange interaction constants at the doped sites compared to the undoped ones. The temperature dependence of the polarization *P* shows a distinctive kink at the Neel temperature $T_{N}$, which shifts to higher temperatures with increasing magnetic field strength *h*. This kink serves as confirmation of the multiferroic nature of KBFO. Furthermore, the introduction of Ru and La dopants results in an overall enhancement of *P*. The temperature dependence of the dielectric constant reveals two peaks at $T_{N}$ and $T_{C}$, which diminish with increasing frequency, indicative of the multifunctional nature of KBFO. Notably, the magneto-dielectric coupling is found to be negative, suggesting that *h* can be effectively employed to control the electric polarization and dielectric constant in KBFO. This fact is important for potential applications where manipulation of these properties is necessary.

Let us emphasize that codoping, for example with Ru and Co, would show a stronger increase in *M* and *P* or decrease in $E_{g}$ than the Ru- or Co-doped compounds. Recently, the codoping effects of different ions on the properties of KBFO were studied in [17]. These effects will be studied in a next paper.

## Data Availability

Data are contained within the article.
